# MDRD vs. CKD-EPI in comparison to ^51^Chromium EDTA: a cross sectional study of Malaysian CKD cohort

**DOI:** 10.1186/s12882-017-0776-2

**Published:** 2017-12-13

**Authors:** Maisarah Jalalonmuhali, Soo Kun Lim, Mohammad Nazri Md Shah, Kok Peng Ng

**Affiliations:** 1 Department of Medicine, University Malaya Medical Centre, 59100 Kuala Lumpur, Malaysia; 2 Department of Biomedical Imaging, University Malaya Medical Centre, 59100 Kuala Lumpur, Malaysia; 3 University of Malaya Research Imaging Centre, University of Malaya, 50603 Kuala Lumpur, Malaysia

**Keywords:** Glomerular filtration rate, MDRD, CKD-EPI, Comparison

## Abstract

**Background:**

Accurate measurement of renal function is important: however, radiolabelled gold standard measurement of GFR is highly expensive and can only be used on a very limited scale. We aim to compare the performance of Modification of Diet in Renal Disease (MDRD) and Chronic Kidney Disease-Epidemiology Collaboration (CKD-EPI) equations in the multi-ethnic population attending University Malaya Medical Centre (UMMC).

**Methods:**

This is a cross-sectional study recruiting patients, who attend UMMC Nephrology clinics on voluntary basis. 51-Chromium EDTA (^51^Cr-EDTA) plasma level was used to measure the reference GFR. The serum creatinine was determined by IDMS reference modified Jaffe kinetic assay (Cr_Jaffe_). The predictive capabilities of MDRD and CKD-EPI based equations were calculated. Data was analysed using SPSS version 20 and correlation, bias, precision and accuracy were determined.

**Results:**

A total of 113 subjects with mean age of 58.12 ± 14.76 years and BMI of 25.99 ± 4.29 kg/m^2^ were recruited. The mean reference GFR was 66.98 ± 40.65 ml/min/1.73m^2^, while the estimated GFR based on MDRD and CKD-EPI formula were 62.17 ± 40.40, and 60.44 ± 34.59, respectively. Both MDRD and CKD-EPI were well-correlated with reference GFR (0.806 and 0.867 respectively) and statistically significant with *p* < 0.001. In the overall cohort, although MDRD had smaller bias than CKD-EPI (4.81 vs. 6.54), CKD-EPI was more precise (25.22 vs. 20.29) with higher accuracy within 30% of measured GFR (79.65 vs. 86.73%).

**Conclusion:**

The CKD-EPI equation appeared to be more precise and accurate than the MDRD equation in estimating GFR in our cohort of multi-ethnic populations in Malaysia.

## Background

The incidence of end stage kidney disease (ESKD) is increasing worldwide. In Malaysia, there were 3167 new cases of ESKD back in 2005 [1]. Over a period of 10 years, the incidence has doubled up to 7055 patients while the prevalence of ESKD was reported as 34,767 cases [1]. The cause of ESKD in our country is still dominated by diabetes mellitus, hypertension and, in part, aging process. Based on the latest US Renal Data System (USRDS), Malaysia is amongst the countries with high incidence and prevalence of ESKD [2]. Therefore, early diagnosis and detection of chronic kidney disease (CKD) is extremely important so that appropriate measures can be taken to impede the progression and the need of early renal replacement therapy. This will subsequently improve cardiovascular outcome and lessen the economic burden of renal replacement therapy.

Glomerular filtration rate (GFR) is the best overall index in assessing kidney function in health and disease. Based on the K/DOQI guidelines, one of the criteria to diagnose CKD is GFR < 60 ml/min/1.73m^2^ [3]. The value of GFR is not just for the diagnosis and early referral to nephrologist but, it is also useful as a guide for the correct drug dosages in general medicine, oncology and to adequately prepare the patients for any contrasted imaging procedure.

The current recommended gold standard for radiolabelled GFR measurement is by using exogenous markers such as iothalamate, Chromium 51 ethylenediamine-tetraacetic acid (^51^Cr-EDTA EDTA) or iohexol. ^51^Cr-EDTA is a well-recognised exogenous marker and it is widely used for the assessment of GFR [4, 5]. However, the use of these exogenous markers is expensive, labour intensive, time consuming and not widely available in our country. Therefore, estimated GFR (eGFR) calculation is important to overcome this problem. The best eGFR equation, ideally, should have lower bias and limits of agreement with greater precision and accuracy. To date, Modification of Diet in Renal Disease (MDRD) and Chronic Kidney Disease-Epidemiology Collaboration (CKD-EPI) equations are widely accepted to be used in clinical practices for GFR estimation [6, 7].

The MDRD equation was developed from a multicentre, controlled trial, to evaluate the effect of dietary protein restriction and strict blood pressure control in CKD population back in the 1990’s [8]. The development of MDRD was to overcome overestimations of eGFR using Cockcroft-Gault equation and creatinine clearance. However, subsequent study has reported that MDRD underestimated eGFR ≥ 60 ml/min/1.73m^2^ [9]. Furthermore, the MDRD equation includes a factor for the black race and the white American. In recent years, in Asian populations, there is a trend to validate this formula with the addition of a racial coefficient. In Chinese and Japanese populations, it has been studied that modifying the racial coefficient for the abbreviated MDRD largely improved the accuracy in estimating GFR in their population [10–12].

CKD-EPI was introduced in 2009 to address some of the limitations from MDRD equation. It was aimed to be as good as MDRD in estimating GFR of <60 ml/min/1.73m^2^ with better estimation of GFR ≥ 60 ml/min/1.73m^2^ [7]. MDRD equation was developed among established CKD patients, thereby limiting its capabilities in estimating GFR in a more ‘normal’ kidney function patient. In contrast, CKD-EPI utilised data generated from the database of National Health and Nutrition Examination Survey (NHANES) among the United States populations. However, there were fewer numbers of patients older than 70 years old and other minorities other than Black recruited in that study. There were few other studies validating CKD-EPI equation performed in Europe ancestry, which was in favours of CKD-EPI [13]. However, this may not be applicable to other populations in the world with diverse background and ethnicity.

Thus, the aim of this study is to compare the performance of the MDRD and CKD-EPI equations in estimating GFR in our multi-ethnic Malaysian population. Furthermore, we would like to assess whether these equations performed differently when assessing GFR in different groups of CKD stages.

## Methods

This is a cross-sectional study recruiting patients in a continuous manner from the University of Malaya Medical Centre (UMMC) out-patient nephrology clinic. Every patient involved, volunteered to be part of this study and had written an informed consent before participation. They must be ≥18 year-old with stable kidney function for at least 3 months prior to recruitment and healthy during recruitment period. Exclusion criteria include acute kidney injury, ESKD, bed-ridden, malnourished, limb amputee and pregnant women. The study was approved by the University of Malaya Medical Center Medical Ethic Committee with IRB reference number 823.5.

Baseline blood investigations including serum creatinine were taken on the day of the procedure. Subsequently, repeated blood samples for GFR measurement were taken from different arms at 2, 2.5, 3 and 4 h following a single injection of ^51^Cr-EDTA. GFR was subsequently calculated using the slope-intercept method and normalised to body surface area (BSA) of the patients. BSA was calculated using formula: BSA = 0.007184 X height^0.725^ x weight^0.425^ [14]. The result was then corrected using Brochner-Mortensen eq. [15]. Serum creatinine was measured on a clinical chemistry analyser with an assay using a modification of the kinetic Jaffe reaction (alkaline picrate reaction). This modified technique was reported to be less susceptible than conventional methods to interference from non-creatinine Jaffe positive compounds [16]. The creatinine assay was adjusted for calibration with the isotope dilution mass spectrometry (IDMS). Estimated GFR using MDRD and CKD-EPI were calculated using the formula in Table 1.

**Table 1:** MDRD and CKD-EPI formula calculations

Formula	Gender	Equations
MDRD	Male	32,788 x Serum Creatinine −1.154 x Age − 0.203 x {1.212 if Black}
	Female	32,788 x Serum Creatinine −1.154 x Age − 0.203 x {1.212 if Black} × 0.742 (Serum Creatinine in umol/L)
CKD-EPI	Male	141 x min (SCr/0.9,1) -0.411 x max (SCr/0.9,1) -1.209 × 0.993 Age x {1.159 if Black}
	Female	141 x min (SCr/0.7,1) -0.329 x max (SCr/0.7,1) -1.209 × 0.993 Age x {1.159 if Black} × 1.018

For comparison of the 2 eGFR methods; bias, precision and accuracy within 30% of the measured GFR (^51^Cr-EDTA) were determined. Bias is defined as, the mean difference between eGFR and the measured GFR. Precision of the eGFR is determined as standard deviation (SD) of the mean difference between measured GFR and eGFR. Accuracy is determined by integrating precision and bias, and it is calculated as the percentage of GFR estimates within 30% of the measured GFR. Bland and Altman scatter plot illustrates the association and limits of agreement between the eGFR and the measured GFR. The analyses were performed using SPSS software (version 20; SPSS Inc., Chicago, IL, USA) and *p*-value of ≤0.05 was considered as significance level.

## Results

### Patient’s characteristics

A total of 113 patients with mean age of 58.7 ± 12.6 years were recruited for this study. Majority of them were male patients (90.2%) and the average body mass index (BMI) was 26.5 ± 4.6 kg/m^2^. The Malays represented 63.7% of the group, while Chinese was 23.0% and Indian 13.3%, reflecting the general racial distribution in the country. Mean serum creatinine was 137.87 ± 76.54 umol/l and the mean measured GFR using ^51^Chromium EDTA was 66.98 ± 40.65.

### Comparison of MDRD and CKD-EPI with measured GFR

Table 2 provides overall results of mean, bias, precision and accuracy for both equations in estimating GFR in comparison to measured GFR. MDRD and CKD-EPI were well-correlated with measured GFR by using Pearson’s correlation with *r* = 0.806 and 0.867 respectively with *p* value of <0.001. Although MDRD had smaller bias, CKD-EPI, clearly, was more precise and accurate in estimating GFR in the overall group.

**Table 2:** Comparison between MDRD and CKD-EPI with measured GFR

GFR	Mean ± SD	p-value	Bias	Precision	Accuracy within 30% of measured GFR (%)
Measured GFR (^51^Cr-EDTA)	66.98 ± 40.65	–	–	–	–
MDRD	62.17 ± 40.40	< 0.001	- 4.81	25.22	79.65
CKD-EPI	60.44 ± 34.59	< 0.001	- 6.54	20.29	86.73

To further assess the performance of both equations, bias, precision and accuracy were determined for different stages of CKD. The patients were divided into 4 stages of CKD except for stage 5, namely; GFR < 30, GFR 30–59, GFR 60–89 and GFR ≥ 90 ml/min/1.73m^2^ based on the measured GFR values (Table 3).

**Table 3:** Bias, precision and accuracy of MDRD and CKD-EPI in different stages of CKD

CKD Stages, (*n* = 113)	Bias		Precision		Accuracy within 30%	
	MDRD	CKD-EPI	MDRD	CKD-EPI	MDRD	CKD-EPI
< 30 (22)	0.7409	0.5136	4.36432	4.16714	86.36	90.91
30–59 (38)	3.1471	2.6763	7.08731	7.17105	84.21	92.11
60–89 (18)	1.6656	1.0444	26.22318	18.04505	77.78	77.78
≥ 90 (35)	13.4449	19.0800	39.52505	29.74621	65.71	82.86

CKD-EPI was found to have lower bias in GFR < 90 ml/min/1.73m^2^, whereas in subgroup of GFR ≥ 90 ml/min/1.73^2^, MDRD had smaller bias than CKD-EPI. This could explain the smaller bias with MDRD in the overall group. However, one needs to interpret this with caution as the number of subjects was small. In terms of precision, both MDRD and CKD-EPI equations performed equally fair in the subgroups with GFR < 30 and 30–59 ml/min/1.73m^2^. However, marked differences can be seen in the subgroups with GFR between 60 and 89 and ≥90 ml/min/1.73m^2^, where CKD-EPI clearly demonstrated better precision in comparison to MDRD. Even though there were some inconsistency regarding bias and precision for both eGFR equations, CKD-EPI outperformed MDRD in terms of accuracy within 30% of measured GFR in subgroups with GFR < 60 ml/min/1.73m^2^.

**Fig. 1:**
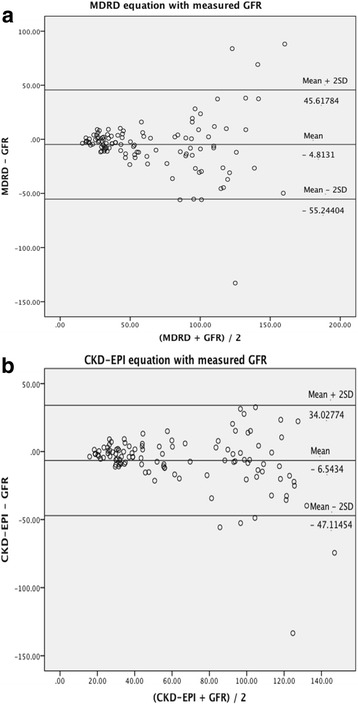
Bland and Altman analysis of GFR estimates. In this analysis, the differences between estimated and measured GFR are plotted against the average of the estimated and measured GFR for each individual patient. 1a. MDRD equation and measured GFR. 1b. CKD-EPI equation and measured GFR

Subsequently, the differences between estimated and measured GFR were illustrated using a graphical technique according to Bland and Altman scatter plot. These figures (Fig. 1a and b) display the span between +2SD and – 2SD of the mean difference (limits of agreement between 2 methods), which represent 95% CI. A smaller limit of agreement was found for CKD-EPI (81.14) as compare to MDRD (100.86).

Table 4 demonstrated that patients might be classified into different stages of CKD depending on the eGFR equations used. Both MDRD and CKD-EPI tend to overestimate GFR in subgroup <30 ml/min/1.73m^2^. One subject was labelled to have CKD stage 5 with GFR < 15 ml/min/1.73m^2^ by both eGFR equations (none when using ^51^CR-EDTA). Interestingly, both MDRD and CKD-EPI performed equally in estimating GFR for CKD stage 3, 4 and 5. MDRD was closer to measured GFR in estimating GFR in subgroup 60–89 ml/min/1.73m^2^, 4.4% better than CKD-EPI. However, the opposite findings was found in subgroup of GFR > 90 ml/min/1.73m^2^, where CKD-EPI was better than MDRD by exactly the same margin.

**Table 4:** CKD staging based on 51Cr-EDTA and 2 different eGFR formulas

CKD staging (ml/min/1.73m^2^)	Measured GFR (^51^Cr-EDTA) n (%)	MDRD n (%)	CKD-EPI n (%)
< 15	0 (0%)	1 (0.9%)	1 (0.9%)
15–29	22 (19.5%)	28 (24.8%)	28 (24.8%)
30–59	38 (33.6%)	37 (32.7%)	37 (32.7%)
60 – 89	18 (15.9%)	19 (16.8%)	14 (12.4%)
≥ 90	35 (31.0%)	28 (24.8%)	33 (29.2%)

## Discussions

The role of GFR estimation in daily clinical practice is unarguably important. It serves as a reasonably excellent ‘substitute’ to the meticulous and costly radiolabelled measurement of GFR, although it may not always be perfect. Comparison among the available GFR estimation formulas in various groups have been done to compensate for the imperfection of these formulas [17, 18]. However, such studies, especially comparison against the measured GFR, are lacking in our multi-ethnic and multi-cultural Malaysian CKD group. With the increasing incidence of ESKD locally and globally, more comprehensive assessments are important for early detection of the population at risk and retard the progression of CKD [1, 2].

As per K/DOQI recommendation, the referral to nephrologist begins at eGFR <60 ml/min/1.73m^2^ [3]. In developing countries like Malaysia, the bulk of patients are taken care of by the primary healthcare doctors in the peripheral hospitals or clinics. It is therefore, extremely important to equip them with the knowledge on GFR assessment and first-line management whenever they come across CKD patients in their practice. The concept of relying on serum creatinine alone should be systematically replaced by eGFR calculation. Practical and ‘user-friendly’ eGFR formula would certainly help to some extent because ‘gold-standard’ assessment of GFR is only available in major or tertiary hospitals. In has been proven, even in the developed countries like the United States, that primary care physicians would recommend earlier nephrology referrals for their CKD patients using eGFR versus serum creatinine [19]. From this current study, CKD-EPI perhaps is better compared to MDRD in identifying the target CKD patients, especially CKD stage 3 and 4.

To date MDRD and CKD-EPI equation are well-accepted to be used globally. In fact, we have also demonstrated that both formulas correlated well with the gold standard ^51^Cr-EDTA. However, the common limitation that both MDRD and CKD-EPI equations have is that the data originated mainly from the Caucasian and African-American population. It has been shown that in Asian population, coefficient factor for MDRD is needed to improve the accuracy of the formula [10–12]. However, identification of specific coefficients for our population is beyond the scope of this present study. Performances of MDRD and CKD-EPI in Asian population have also been assessed in prior studies, and not in our multi-ethnic predominantly Malay population [20–22]. In those studies, CKD-EPI consistently performed better than MDRD in estimating GFR just like what we discovered in our group.

This study is simply to compare the suitability of these 2 commonly accepted eGFR estimates formula. The findings are aimed at guiding the general healthcare providers in choosing the better eGFR formula for their patients. In our study, CKD-EPI formula seems to perform better than MDRD formula. Across all stages of CKD, CKD-EPI has greater precision and accuracy within 30% with lower limits of agreement.

Based on the previous data, we know that MDRD underestimates GFR when the eGFR ≥60 mls/min/1.73m^2^. Similar pattern can be seen here in our study with the study reported by Stevens et al. earlier in 2010 [10]. Although the eGFR between 60 and 89 mls/min/1.73m^2^ showed that MDRD seems to have similar classification with 51Cr-EDTA results, this was not true when the GFR increased further to ≥90 mls/min/1.73m^2^. In this group, we identified more patients with supposedly ‘normal’ GFR as compare to MDRD. This certainly has the potential to avoid unnecessary referral to nephrologist or intervention.

There are few limitations of the study to note. 1) Comparatively small sample size 2) Predominantly male patients – due to the recruitment bias, males tend to be more forthcoming 3) Non-randomised recruitment of the patients. Therefore, we may not be able to draw any strong conclusion from a study of this nature. Nevertheless, this is among the first to critically compare between MDRD and CKD-EPI in our unique multi-ethnic Malaysian cohort using ^51^Cr-EDTA as reference gold standard for GFR. The use of an exogenous marker such as ^51^Cr-EDTA for the measured GFR measurement is particularly distinct as no study such as this has ever been conducted in Malaysia. The results might have an impact on the use of CKD-EPI formula in estimating GFR at the out-patient settings.

## Conclusion

We conclude that CKD-EPI equation had smaller bias and greater accuracy than the MDRD equation in estimating GFR of <60 ml/min/1.73m^2^. Although accuracy was the same for subgroup GFR between 60 and 89 ml/min/1.73m^2^, CKD-EPI had smaller bias as well as better precision. In estimating GFR of >90 ml/min/1.73m^2^, CKD-EPI had greater precision and accuracy than MDRD, but was more biased.

## Abbreviations

4-MDRD: Modification of diet in renal disease
^51^Cr-EDTA: 51 chromium ethylenediamine tetraacetic acid
BSA: Body surface area
CGBSA: Cockcroft-Gault corrected for body surface area
CKD: Chronic kidney disease
CKD-EPI: Chronic Kidney Disease Epidemiology Collaboration
CKD-EPIcr: CKD-EPI creatinine
CKD-EPIcys: CKD-EPI cystatin
ESKD: End stage kidney disease
GFR: Glomerular filtration rate
MDTR: Malaysian Dialysis and Transplant Registry
mGFR: measured glomerular filtration rate
USRDS: US Renal Data System
